# ‘Incantatory’ governance: global climate politics’ performative turn and its wider significance for global politics

**DOI:** 10.1057/s41311-020-00250-8

**Published:** 2020-05-27

**Authors:** Stefan C. Aykut, Edouard Morena, Jean Foyer

**Affiliations:** 1 Fakultät WISO | FB Sozialökonomie, Universität Hamburg, Welckerstr. 8, 20354 Hamburg, Germany; 2 University of London Institute in Paris (ULIP), 9-11 Rue de Constantine, 75340 Paris Cedex 07, France; 3 IHEAL/CREDA, Campus Condorcet, Batiment de recherché sud, 5 cours des humanités, 93233 Aubervilliers, France

**Keywords:** Global climate governance, Paris agreement, New public management, Narratives, Performativity

## Abstract

The 2015 Paris agreement represents a deep-rooted change in global climate governance. While existing scholarly assessments highlight central *institutional* features of the Paris shift, they tend to overlook its *symbolic and discursive* dimensions. Our analysis shows that the Paris architecture combines two core elements: an iterative pledge and review process to stimulate global climate action, and a ‘performative’ narrative aimed at aligning actors’ expectations on the prospect of a low-carbon future. We therefore suggest calling it an *incantatory* system of governance. We then examine the origins of the new approach and find that the rise of ‘soft law’ approaches and communicative techniques in global climate governance are both indicative of a broader process: the entry of management culture in international organisations. Against this backdrop, we examine the prospects, limitations and caveats of the new approach and discuss its wider implications for global politics.

## Introduction

The Paris agreement adopted in December 2015 is widely considered as a major breakthrough in global climate governance, with the potential of becoming a blueprint for other governance arenas (Jordan et al. 2018). And yet, just 2 years after its adoption, it was already in jeopardy when US President Trump announced on 1 June 2017 his intention to withdraw from the treaty. The decision completely paralysed negotiations at the UN climate summit COP23 in Bonn in November of that same year. Interestingly, however, the atmosphere was very different at the ‘Bonn Zone’, an area dedicated to non-state and sub-state climate efforts and just a few hundred metres away from the official conference space. A highlight of the ‘Bonn Zone’ was the launch of the #WeAreStillIn coalition. Under the leadership of billionaire philanthropist, former New York City mayor and UN special envoy for climate action Michael Bloomberg, as well as California governor Jerry Brown, the coalition brought together American cities, states and businesses committed to fulfilling the US’s national emission reduction commitments through bottom-up action. The mood was similarly upbeat at the One Planet Summit in Paris a month later. Convened by French President Emmanuel Macron to mark the COP21’s second anniversary, the Summit provided business and NGO leaders, representatives from international organisations and national and multilateral development banks, heads of state and government, philanthropists and mayors with an opportunity to both reassert their commitment to the Paris agreement and to announce new measures for its implementation.

The ‘Bonn Zone’ and One Planet Summit are revealing of the current state of global climate governance. They are symptomatic of more deep-rooted shifts in its organisation, in the levels of engagement, in the actors involved, and the mechanisms through which it operates and produces effects. Global climate policy is now understood as a process that transcends the United Nations Framework Convention on Climate Change (UNFCCC), and of which transnational initiatives and private governance schemes constitute an integral part (Moncel and van Asselt 2012). Furthermore, it is no longer aimed at the production and enforcement of binding reduction targets for states, but builds on a flexible ‘pledge and review’ system combining voluntary pledges by public *and* private actors alike, and binding reporting and transparency rules for states (Keohane and Oppenheimer 2016). Taken together, these changes have been described as a shift away from a ‘regulatory’ and towards a ‘catalytic and facilitative model’ of global governance (Hale 2016). While such assessments highlight central aspects of the Paris shift, they also contain significant blind spots. The bulk of stand-alone articles and special issues on post-Paris climate governance focusses on negotiation dynamics and outcomes,^1^ the interpretation of the agreement’s legal dispositions,^2^ or institutional innovations in the post-Paris process (Jordan et al. 2018). In doing so, such analyses tend to overlook an important feature of the new governance regime: its symbolic and discursive dimensions. As illustrated by the examples above, the post-Paris process conveys a central role to the emission of ‘signals’ and the creation of ‘momentum’ for climate action, through carefully orchestrated global moments such as the One Planet Summit and Climate Action Summits and highly publicised private initiatives like #WeReStillIn. In other words, in this new governance, performances, symbols and narratives appear to be just as important as the production of rules, institutions and instruments.

We therefore suggest calling the new approach an *incantatory system of governance.* On a general level, the notion of ‘incantation’ points to the ritualised and repetitive dimensions of global climate governance, with its annual meetings and recurring calls to urgency and action (Little 1995), as well as to the theatrical dramaturgy of climate summits and their filiation to the ‘society of spectacle’ (Death 2011). More specifically, it permits to capture what we believe constitutes a distinctive feature of the new approach: the fact that communicative and symbolic devices are explicitly recognised, by its architects and promoters, as *core instruments* in the agreement’s implementation. A central element in this context is the grand narrative of an ongoing ‘planetary transition’ to a decarbonised world economy, which is crafted and circulated by key governance actors. By using the notion of incantation, we also wish to engage a discussion on the origins and wider significance of this governance shift. In an increasingly fragmented (Biermann et al. 2009), marketised (Newell and Paterson 2010) and privatised (Park et al. 2008) global governance landscape, ‘soft law’ approaches resting on voluntary commitments (Abbott and Snidal 2000), indicators and best practices (Merry 2011) have been on the rise over the last decades. The Paris shift fits within this broader set of transformations, inspired by the adoption of New Public Management (NPM) methods in international organisations. We suggest that these two dynamics—the weakening of legal and regulatory frameworks, and the inflationary use of communicative devices—can be understood as two sides of the same phenomenon: the importation of a business culture in global governance. Finally, the notion of incantation points to the need to renew the methods with which we study global climate governance. Our aim is not to present the new approach as ineffective per se, but to understand how it plays out *in practice*, and better appreciate its prospects, risks and caveats. This requires examining the role of rituals, symbols and discourses in global governance, analyse how they produce effects and study how they relate to, or combine with, more traditional governance methods—such as the negotiation of legal documents and the action of international organisations. In line with collaborative event ethnography (Campbell et al. 2014), our analysis is therefore based on repeated collective observations of different spaces of global climate governance, particularly during the 2015 Paris COP. There, we studied the circulation of people and documents, practices of text production and editing, the role of diplomatic rituals and political performances, as well as civil society mobilisations, scientific events and business happenings (Aykut et al. 2017). We also analysed how philanthropic foundations and think tanks shaped the ‘road to Paris’ and the discursive context surrounding COP21 (Morena 2016). In this paper, we connect the findings of these different lines of research. Drawing on discourse analysis (Bäckstrand and Lövbrand 2007), we also reconstruct the narratives circulated by promoters of the new governance approach. That being said, the article’s primary goal is to advance a broader conceptual argument. The empirical material serves to shed light on our argument rather than provide a comprehensive, rigorous analysis of *one* conference or *one* discourse.

## Performative iterations: an anatomy of the Paris approach

The Paris approach introduces a series of institutional innovations. It marks a transition from a ‘regulatory’ approach to global climate governance, with detailed rules and obligations that apply to developed states, to a ‘hybrid’ system that both combines voluntary submissions and binding review cycles for all states and associates a wider range of stakeholders. However, in the eyes of its architects and main proponents, the new approach does not only rest on new institutions, it also centrally relies on new discursive and symbolic elements.

### An iterative process to ‘facilitate’ and ‘orchestrate’ global climate action

Instead of legally binding reduction targets and sanctions for non-compliance, the governance framework laid out in the Paris agreement is based on the submission and review of freely determined policy pledges, or Nationally Determined Contributions (NDCs). However, the approach also differs significantly from purely voluntary systems. On the substantive side, it sets two long-term temperature goals: keeping global warming ‘well below’ 2 °C and ‘pursuing efforts’ to stay below 1.5 °C. The COP decision also sets out the figure of 100 billion USD per year towards developing countries’ adaptation and emissions reduction efforts. Apart from the 1.5 °C target, these figures had already been laid out in the contested Copenhagen Accord in 2009. Accordingly, the Paris agreement’s main innovations are procedural, rather than substantive (Oberthür and Bodle 2016). An ‘enhanced transparency framework’ is set up to ensure the publicity and comparability of NDCs;^3^ a ‘global stocktake’ is scheduled every 5 years to collectively evaluate the adequacy of national efforts; based on this assessment, countries are expected to ‘ratchet up’ their pledges in line with the agreement’s long-term goals. In sum, the Paris framework establishes legally binding obligations of *conduct*, but no obligations of *result* (Bodansky 2016). Its implementation has been described as a ‘two level game’ in which the capacity of domestic civil societies to exert pressure on their governments plays a decisive role (Keohane and Oppenheimer 2016). The Paris architecture is therefore understood by its proponents as an iterative process, in which ‘the many interdependent parts […] interact in mutually facilitative ways’ (Hale and Roger 2014: 535).

The agreement also broadens the scope of stakeholders that participate in global climate governance. In addition to developed countries, developing countries as well as private and subnational actors are encouraged to submit emission reduction pledges. In this respect, Paris not only marks a historic break with the North–South divide in global climate politics; it also confirms the rise of ‘private authority’ and corporate self-regulation in global governance (Pattberg 2005; Andonova 2010). From centrepiece of a unified and centralised climate regime, the UNFCCC is now considered as only one of many elements that collectively make up a broad landscape of ‘transnational climate governance’ (Betsill et al. 2015; Bulkeley et al. 2014). In the lead-up to the Paris conference, climate governance scholars reassessed the UNFCCC’s role.^4^ They encouraged it to take on an ‘orchestrating’ function for climate action by states, as well as businesses, cities, regions and federated states (Abbott and Snidal 2009; Moncel and van Asselt 2012; Hale and Roger 2014). Orchestration is thereby defined as an ‘indirect mode of governance that relies on soft inducements’, as the orchestrator ‘works through like-minded intermediaries, catalysing their formation, encouraging and assisting them and steering their activities through support and other incentives’ (Abbott 2018: 189). An oft-cited example is the UNFCCC’s Non-State Actor Zone for Climate Action (NAZCA), an online platform launched in 2014 ‘where actors from around the globe—countries, regions, cities, companies, investors and other organisations—can display their commitments to act on climate change.’^5^ To further encourage transnational climate action and link it to the UN process, the UNFCCC also promoted ‘High-Level Champions’ for climate action. The ‘Champions’—usually personalities from the business, political and cultural spheres—put their professional networks and celebrity in the service of climate action. In return, the UN arena provides social prestige and symbolic recognition to these individuals.

### A mobilising narrative to align stakeholders’ expectations

Initiatives such as NAZCA portal or the High-Level Champions are envisioned as more than mere appendages to national efforts. They are a constitutive ‘fourth pillar’ of global climate governance alongside mitigation, adaptation and climate finance, intended to ‘galvanize’ and ‘catalyse’ global climate action (Hale 2016). The underlying image is that of a virtuous cycle, in which experiences of past cooperation create trust and confidence among actors and alter their future preferences (Bang et al. 2016). The concept of ‘catalytic cooperation’ (Hale 2018) neatly captures this idea. It rests on the claim that global mitigation efforts have wrongly been portrayed as a classic case of a prisoner’s dilemma. Instead, it is argued that climate action entails first mover benefits for pioneers and increasing returns as the number of followers increases. This may lead to normative change through ‘norm cascades’ and ‘tipping points’ that transform the incentive structure and hence the nature of the problem. Hence, ‘the entire purpose of a catalytic regime’ like the Paris agreement ‘is to shift actors’ preferences over time in favour of cooperation’ (Hale 2018: 22). Given the importance of norms, trust and preferences in this governance setup, however, surprisingly little attention have been paid in the literature to global climate governance’s symbolic and discursive dimensions. Indeed, the defining feature of contemporary climate governance is that signals, narratives and performative moments are *at its core*. This is explicitly recognised by key proponents of the new approach. Laurence Tubiana, special envoy of the French Presidency to the COP21 negotiations and one of the architects of the Paris agreement, presents the treaty as a ‘self-fulfilling prophecy’, whereby positive narratives ‘by producing a convergence of rational anticipations […] contribute as much to change as the agreement itself.’^6^ The main objective of post-Paris climate governance is no longer the production of new legal norms, but the alignment of state and non-state actors’ expectations on the prospect of a low-carbon future. The ‘signals’ and ‘momentum’ generated by the governance process underpin the voluntary architecture of the agreement.

## Fifty shades of soft: fostering a new institutional framework

While the origins of the bottom-up approach in global climate governance are often traced back to the 2009 Copenhagen climate conference (COP15), fully capturing how and why it came about, and what constitutes its specificities, requires us to go further back in time. Indeed, voluntary approaches have been part of the discussions since the beginning of climate talks in the 1990s. We also need to expand our horizons to other areas of global politics, as the approach adopted in Paris echoes a wider ‘managerial turn’ in global politics.

### The ups and downs of voluntary approaches in climate negotiations

Prevailing accounts of the Paris shift tend to focus on dynamics *within* the climate regime. And indeed, the idea of a voluntary framework to coordinate the global mitigation effort historically emerged in the run-up to the 1992 Rio conference. At that time, the EU favoured a ‘targets and timetables’ approach based on binding reduction commitments for industrialised countries. The US administration criticised the proposal as overtly ‘top-down’ and ‘rigid’, arguing that climate governance should involve a more flexible ‘bottom-up’ approach (Bodansky 1993: 514). As a compromise solution, Japan suggested in July 1991 a pledge and review system combining voluntary country submissions and an international review process to track implementation. However, the targets and timetables approach ultimately won over in Kyoto in 1997 (Damian 2014). The voluntary approach resurfaced in the run-up to the Copenhagen conference, where countries negotiated on a successor treaty to the Kyoto protocol. Two years earlier, the so-called ‘Bali Action Plan’ had introduced the concept of ‘Nationally Appropriate Mitigation Actions’ as a means of getting developing countries to contribute to the mitigation effort. The idea was to encourage emerging economies to make voluntary pledges that would be subject to measurement, reporting and verification (MRV). This, it was hoped, would trigger an incremental process, whereby pledges would progressively be strengthened and ultimately be converted into binding commitments. In the midst of the Copenhagen collapse, however, the voluntary approach was ultimately extended to the global North as well. Intended Nationally Determined Contributions (INDCs) were introduced as a compromise solution between ‘Nationally Appropriate Mitigation Actions’ and the quantified emissions reduction objectives that applied to developed states under the Kyoto protocol.^7^ The origins of the voluntary approach can therefore be traced back to the early years of the climate regime. There are, however, important differences between the initial proposals and the Paris approach. These relate not only to the specific ways in which the Paris agreement combines binding and non-binding elements, but also to the broader global setting in which the new climate governance is embedded. This setting differs significantly from the early 1990s.

### Management culture’s incursion into global governance

In the post-Cold War context of the 1992 Rio conference, the widely held view was that global governance unfolds mainly through global institution building and the gradual strengthening of international law (Levy et al. 1995; Zangl and Zürn 2004). Advocates of pledge and review in the early climate negotiations could therefore frame such a system as a first, incremental step towards more substantial commitments later on (Bodansky 1993: 486). This argument appears less plausible today, as the voluntary turn in climate governance coincides with major transformations in global governance. The global diffusion of ‘regulatory capitalism’ (Lévi-Faur 2005) and the rise of ‘private authority’ (Hall and Biersteker 2002; Pattberg 2005) challenge the long-standing supremacy of states and international organisations in global affairs. In a multi-actor world (Kaul et al. 1999), global governance no longer unfolds through state-led multilateralism alone, but also through forms of ‘transnational regulation’, ‘hybrid governance arrangements’ (Graz 2006; Andonova 2010) and networks of corporate self-regulation (Müller and Cloiseau 2015; Short 2012).

These transformations had as a corollary the introduction of new governance methods, which originated in the business sector. In the 1970s, new management techniques such as Total Quality Management aimed to provide firms with ‘remote control’ over their increasingly transnational production chains, through a circular procedural sequence of goal-setting, reporting and auditing (Power 1999). These techniques inspired a range of national administration reforms during the ‘managerial moment’ of the 1980s and 1990s (Kroeze and Keulen 2014; Pollitt and Bouckaert 2011), before spreading to the global level. Corporate Social and Environmental Responsibility (CSER) schemes, which rely on a similar circular process of pledging, reporting and review, contributed to this dissemination (Zumbansen 2006; Crane et al. 2008). Through partnerships in such schemes, members of NGOs think tanks and international organisations were progressively ‘acculturated’ to business methods, practices and vocabulary (Conley and Williams 2008: 14, 15). The spread of CSER is also associated with a process of private ‘re-regulation’ (Logsdon and Wood 2002; Conley and Williams 2011), whereby businesses became recognised sources of policy proposals at the international level (Müller 2013). International organisations followed suit over the next decades and increasingly adopted ‘soft’ and ‘experimental’ governance methods (Sabel and Zeitlin 2012; Eckert and Börzel 2012). The Millennium Development Goals (MDGs), the UN Global Compact and the EU’s Open Method of Coordination—three processes launched at the turn of the millennium—constitute paradigmatic examples for this trend. All three combine the definition of common goals, decentralised implementation methods and collective review and benchmarking mechanisms. In addition to coinciding with a broader ‘managerial turn’, international organisations’ adoption of more flexible governance modes also signals their increasing difficulty to develop and enforce binding rules on states (Hale et al. 2013). Hence, the Open Method of Coordination was launched in response to critiques of the EU’s overly centralised power structure (Regent 2003; Schout et al. 2010), while the MDGs came on the back of more than a decade of structural adjustment programmes that spurred growing resistance among developing countries (McArthur 2014; Shawki 2016). The direct consequence of these evolutions is a shift in the normative horizon of global governance. If international relations scholars could still claim in the 1990s that the ‘main purpose’ of international regimes was ‘to harmonize national legislation or to establish rules that can be applied by and to states’ (Zartman 1994: 6), this no longer pertains to this new type of governance arrangements. From a system organised around the production of legal documents to be transposed into national law, global governance shifted towards a system grounded on the definition of shared goals, voluntary commitments by state and non-state actors, and global review and monitoring processes.

### Non-state actors as brokers for a bottom-up approach

While UN climate governance was somewhat of a latecomer in adopting the new governance modes, it had been affected by these broader trends well before the Copenhagen and Paris conferences. Since the turn of the millennium, a new ‘transnational climate governance landscape’ (Bulkeley et al. 2014) progressively took root through the emergence of Corporate Social Responsibility schemes (Bulkeley and Newell 2010: 119), transnational city networks (Betsill and Bulkeley 2004) and corporate carbon trading systems (Bernstein et al. 2010). In the climate diplomacy space, this evolution was closely scrutinised and promoted by a well-experienced and well-connected group of diplomats, NGO, foundation and business representatives, climate policy and communications experts in close contact with the UNFCCC Secretariat and key Parties to the Convention (Morena 2016). Bringing together individuals with a history of involvement in the international climate diplomacy space—through initiatives like the Global Call for Climate Action (GCCA) or Project Catalyst, or informal networks such as the Croissant Conspiracy^8^ or the Lionesses^9^—, the International Policies and Politics Initiative (IPPI) provides a telling example of how non-state actors strategically mobilised to orientate the international climate debate. Participants in the Initiative’s mid-2013 ‘lake Tornow’ meeting close to Berlin include representatives from foundations (ECF, CIFF, Vasuda), development NGOs (Oxfam, Care International), environmental NGOs (Greenpeace, WWF), campaign networks (CAN international, 350.org, Avaaz, GCCA), business networks (The Climate Group), think tanks (E3G, WRI, UCS, Ecofys, Track0, IDDRI, Germanwatch, Grantham LSE) and strategic communications (Climate Nexus) (Morena 2016: 118). Launched in April 2013, and building on a 2011 strategy document produced by the European Climate Foundation (ECF), IPPI’s purpose was to deliver ‘a strong climate regime’ that ‘[fostered] bottom-up action [anchored] in top-down elements’ (European Climate Foundation 2011: 3).

For participants in IPPI, the failure to reach an agreement in 2009 was a direct consequence of stakeholders’ disregard for wider political and non-state actor dynamics and their influence. Experts from the think tank Third Generation Environmentalism (E3G, founded in 2004), for instance, suggested that the Copenhagen collapse had shown that ‘climate diplomacy has shifted from a relatively narrow focus on the UNFCCC process, to a more complex and wider discipline that now engages new constituencies and embraces broader geopolitical discussions’ (Mabey et al. 2013: 6). As Johannes Meier, CEO of the European Climate Foundation (ECF, founded in 2008) explains, experts and activists had failed to recognise that change happens ‘in rather oblique and non-linear ways’ and that there is a ‘need to pay more attention to politics and even to the polity’ (Meier 2015). In its 2011 strategy document, ECF further argues that ‘the radical policy change that will be required’ entails moving not only policy-makers, but ‘society as a whole, from the progressive to the conservative, right to left, engaged and disinterested’ (European Climate Foundation 2011: 4). The new priority in the lead-up to Paris was therefore to stimulate actions at multiple levels and locations, both within and beyond the UNFCCC, and involve a wide range of stakeholders, to create the conditions for a new type of global climate agreement. The idea was to deliver an agreement that combined a long-term goal that sends ‘a clear signal to policy makers, businesses, investors and the public that the low-carbon climate-resilient economy is inevitable’ (Morgan et al. 2014: 4), with ‘bottom-up’ commitments that are regularly updated and subject to robust transparency and accountability provisions. This, it was suggested, would enable climate diplomacy to use the ‘groundswell’ of ‘nonstate action’ to ‘reinvigorate’ global climate governance (Chan et al. 2015). Through these and similar proposals in the run-up to Paris, climate policy experts and representatives from think tanks, philanthropic foundations and environmental NGOs successfully positioned the pledge and review approach as a credible and pragmatic alternative to the legally binding, top-down system that had prevailed up to Copenhagen.

## Parole, parole, parole: narratives and signals as tools of governance

Critical governance scholars and ethnographers of global institutions have long argued that discourses, narratives and symbols constitute key elements in the making of global orders and pointed to the importance of rituals and performances in global mega-events like UN climate summits. And indeed, beyond the 12-page treaty and accompanying 20-page COP decision, the Paris COP also gave birth to the mobilising narrative of an ongoing ‘planetary transition’ to a low-carbon economy. The making of this narrative can be traced back to the aftermath of COP15 in Copenhagen, when the production and dissemination of discursive frames became a central concern for climate governance actors. In the process, communication practices became a key strategic tool for the architects of the Paris approach.

### Discourses, rituals and performances in global environmental governance

Making sense of the contemporary transformations of global climate governance requires an analytical vocabulary that adequately captures its discursive and symbolic dimensions. This points to at least two existing lines of research. First, research on discourses and norms highlights the constitutive power of language, knowledge and ideas in global environmental governance (Bernstein 2001; Oels 2005; Pettenger 2007; Hughes and Paterson 2017). Bäckstrand and Lövbrand (2006, 2016), for instance, show how three broad discursive formations—‘ecological modernization’, ‘green governmentality’ and ‘civic environmentalism’—distinctly shaped global climate politics in the post-Kyoto and post-Copenhagen eras. Global climate discourses also extend beyond the realm of UN climate diplomacy. They have disciplining effects on the everyday and participate in the creation of subjectivities (Paterson and Stripple 2010). They provide ‘discursive hooks’ to actors seeking entry into the climate arena (Allan 2018) and enable strategies of ‘climate bandwagoning’ (Jinnah 2011). Moreover, their circulation contributes to a ‘climatisation of global debates’, whereby issues formerly unrelated to climate policy are increasingly scrutinised through a ‘climatic lens’ (Aykut et al. 2017; Oels 2012). UN summits, which attract new actors and issues into the climate arena, play an important role in this progressive extension of the thematic scope and symbolic reach of climate governance. This resonates with a second line of research which focuses on the symbolic and performative dimensions of global environmental summits (Blühdorn 2011; Campbell et al. 2014). Ethnographer Paul Little (1995) provides a fascinating account of the role of performances and rituals at the 1992 Rio conference. Analysing the endless litany of speeches by heads of state and government during the opening ceremony, he shows how these conveyed to the respective home audiences the idea that ‘world leaders’ were best suited to address global problems. Death (2011) makes a similar argument in a foucauldian study of ‘theatrical techniques’ at the 2002 Johannesburg and 2009 Copenhagen summits. ‘Environmental summitry’, he argues, has come to constitute a ‘distinct technology of government’. Despite being unsuccessful in terms of negotiations, the two summits constituted attempts ‘to inspire and conduct the self-optimisation of the watching global audience’. For these authors, global mega-conferences cannot be reduced to formal negotiation outcomes; they are also important loci for the production of meaning, through the emission of signals, frames and narratives.

### Crafting and circulating the grand narrative of a ‘planetary transition’

Such perspectives permit to shed new light on the discursive context of the pre-Paris process. Indeed, Copenhagen also marks the start of a new ‘positive’ narrative around climate change, which would come to form a core feature of the new climate governance. For the group of stakeholders mentioned above, Copenhagen had not only been a diplomatic fiasco, but also a failure in terms of communication. It had effectively failed to shape the overall narrative on climate change in a positive way (Morena 2017: 107, 108). Too little attention had been paid to the symbolic and discursive dimensions of climate diplomacy. To succeed, the Paris conference therefore had to send ‘unambiguous signals that the world will shift its economic and social activity toward more climate-friendly and sustainable pathways’ (Oberthür et al. 2015: 1). To do this, a range of individuals were mobilised and tools were created to ensure that stakeholders in the climate debate sent the right message to the right audience at the right time (Morena 2016). Communications efforts were orchestrated by discreet ‘unbranded’ initiatives such as the Global Strategic Communications Council (GSCC) or Climate Briefing Service (CBS) whose communications experts ‘[coordinated stakeholder] voices at the national and international levels to help shape the national offers as they are being drafted and the thinking around the international agreement’.^10^ They focused their communications efforts on global and national climate-relevant ‘moments’ leading-up to the Paris conference; from G7 and G20 Summits, to the Rio + 20 conference (2012) and associated green growth/green economy agenda, to China’s adoption of its new 5-year plan, to the launching of climate-related reports (IPCC reports, New Climate Economy report, UNEP Emissions Gap reports…). These communications efforts mobilised a wide range of stakeholders, from climate ‘outsiders’ active on the margins of the official negotiation process to climate ‘insiders’ working closely with parties to draft a new treaty (de Moor et al. 2017; Newell 2000). Christiana Figueres, UNFCCC Executive Secretary at the time, played a key role in these efforts. She provides a fascinating account of her intense lobbying work for a climate agreement in a recent *Nature* commentary. Her primary task, she contends, consisted in spreading optimism:

I immediately realized that, before we could consider the political, technical and legal parameters of an eventual agreement, I had to dedicate myself to changing the mood: there could be no victory without optimism. I decided to set a clear intention: even if we did not know precisely how, a global deal would emerge, simply because it was necessary. It was that contagious frame of mind that led to effective decision-making, despite the enormous complexities under which we were operating. When the Paris agreement was achieved, the optimism that people felt about the future was palpable – but, in fact, optimism had been the primary input. (Figueres 2020)

Among the groups that actively promoted a new climate narrative were also progressive business interests like the We Mean Business coalition^11^ launched at the 2014 NYC Climate Week (Benabou et al. 2017). In its first report *The Climate Has Changed*, the coalition argues that ‘the transition to a low-carbon economy is already happening’ (We Mean Business 2014: xiv) and attempts to demonstrate that ‘ambitious climate action makes business sense’ (*Ibid*.: viii). The transition is depicted as a dynamic, polycentric process where ‘bold business action’ and ambitious policy-making are mutually reinforcing (*Ibid*.: vii). A follow-up publication *Shaping a Catalytic Paris Agreement* contains a detailed proposal for a new climate treaty (We Mean Business 2015). According to the authors, such an agreement should combine voluntary and binding elements to ‘[create] an inclusive enabling environment for all stakeholders—including business’ and fix an ambitious temperature target to ‘send a political signal that long-term decarbonisation is inevitable’ (*Ibid*.: 2). In other words, its purpose would be largely *symbolic*. By further substantiating the narrative of an ongoing and unavoidable low-carbon transition, the successful adoption of an ‘ambitious’ agreement would encourage low-carbon efforts by businesses, investors and citizens. This would in turn generate momentum for more ambitious national policies, thereby setting in motion a self-reinforcing process towards decarbonisation. As former US Secretary of State John Kerry explains in the *Rolling Stone*:

If 150 nations are taking it seriously and setting targets, even if they don’t make them, that will generate massive investment and a huge amount of private-sector activity […] And then you have to hope that somebody comes up with clean-energy technology, which makes it competitive with fossil fuel, and then, boom, you get your low-carbon economy.^12^

In the lead-up, during and on the back of COP21, the agreement’s core architects set up an elaborate communications campaign whose purpose was to shape a new climate narrative centred on three elements: the low-carbon transition is already underway; it presents unprecedented economic opportunities, and its successful implementation rests on the cooperation of actors from all sections of society. This, it was believed, would generate ‘momentum’ around the ‘Paris moment’, and more generally the benefits of decisive climate action.

### Upholding the ‘Paris momentum’

For the architects of the Paris approach, narratives and signals were not only key to achieving a positive outcome at COP21; they are equally important in the implementation of the Paris agreement. For Laurence Tubiana, the post-Paris process ‘[is] all about momentum.’^13^ Christiana Figueres (2020) urges all stakeholders ‘to move firmly into a state of stubborn optimism’ and to ‘conceive of success and take immediate steps towards it’. Following the adoption of the Paris agreement and its subsequent ratification and entry into force, a priority for its main proponents was therefore to keep the ‘Paris prophecy’ alive in the hope that this would lead stakeholders to ramp up their levels of ambition in the lead-up to the next global stocktake in 2020. Forging the right narrative and controlling the discursive context of global climate governance thereby become key concerns. In the final chapter, ‘A New Story’ from their book, *The Future We Choose*, C. Figueres and Tom Rivett-Carnac describe this task as follows:

Right now, the predominant stories we are telling ourselves about the climate crisis are not very inspiring. But a new story can reinvigorate our efforts. When the story changes, everything changes (Figueres and Rivett-Carnac 2020: 158).

The purpose of climate summits changes accordingly. In the post-Paris period, negotiations increasingly lose their pivotal role. Instead of focusing on the arduous and conflict-ridden process of political bargaining, rituals and performances occupy centre stage. ‘The ideal COP would send a positive signal(s) to the international community, including investors, regarding the Parties’ and other stakeholders’ direction of travel’ writes Susan Biniaz (2020: 11), lead climate lawyer for the U.S. State Department from 1989 to 2017 and another key actor in Paris. In a growing number of high-level and highly mediatised climate action summits, the UNFCCC now takes on the role of ‘travelling salesman’ for ambitious climate action. COPs or Climate Action Summits are essentially about communicating on the urgency of the climate crisis, highlighting the economic and social benefits of climate action and showcasing existing efforts—especially corporate climate action—to address the crisis (Aykut et al. 2020). Hence, while rituals, discourses, theatrical techniques and political performances have always played an important role in global politics more generally, the post-Paris climate governance stands out. Whereas in other governance arenas their role tends to be understated or played down, in the climate arena, communicative and symbolic elements are explicitly recognised as core instruments in the implementation of the Paris agreement.

## Incantatory governance: prospects, risks and caveats of the new approach

We suggest the term ‘incantatory governance’ to characterise this new approach. In so doing, we aim to highlight both the iterative, cyclical process created by the Paris agreement’s review mechanism and the central role of performative narratives and signals in the post-Paris setup. As pointed out earlier, our intention is not to dismiss the approach as ‘merely’ symbolic and therefore ineffective. Ethnographic research shows that incantatory rituals can produce real-world effects and fulfil important social functions. Claude Lévi-Strauss, for instance, famously investigated the ‘pragmatic effectiveness of symbols’ in shamanistic cure (Muniesa 2014: 21). The repetitive utterance of words and mobilisation of symbols, he writes, ‘provoke[s] an experience’, which can produce therapeutic effects (Lévi-Strauss 1949: 21). An increasing body of research shows that modern institutions also heavily rely on symbols, rituals and narratives: storytelling and drama constitute key features of contemporary management culture (Czarniawska 1997), while ‘fictional expectations’ shape the functioning of capitalist systems (Beckert 2016). Accordingly, Death (2011: 9–10) criticises what he terms the ‘anti-theatrical prejudice’ in social science scholarship. ‘Symbolic aspects of summitry are not sideshops’, he contends, ‘but essential to the manner in which summits govern the conduct of global politics’. Instead of opposing ‘symbolic’ politics to a hypothetical ‘real’ politics, we should accept that symbols and narratives form part and parcel of contemporary liberal governmentality (Blühdorn 2007; Death 2011). The imminent conclusion of the regime building process therefore represents a critical juncture not only for UN climate governance, but also for social science research. What are the prospects, risks and caveats of the new approach? As the focus shifts from negotiation to implementation, a new chapter opens for the UNFCCC and its annual COPs. While a thorough assessment of the effectiveness of the new governance approach would be premature, developments since the Paris COP point towards two main issues with the new approach.

### Governance as symbolic struggle, and the risk of ‘virtuality’

President Donald Trump’s decision in June 2017 to withdraw from the Paris agreement represented a severe test for the post-Paris process. Given the historical responsibility and political weight of the USA, the decision weakened the UNFCCC as the central forum of global climate governance. By sending a very negative signal, the US administration’s retreat also threatened to undermine the ‘Paris prophecy’, which, as we have shown, forms a crucial part of the post-Paris climate governance framework. To uphold the momentum, it therefore became essential to show that the international community—state and non-state actors alike—was still committed to the goals laid out in the Paris agreement, with or without US federal support.

In response to Trump’s decision, the international climate community coordinated a series of high-profile initiatives. Notable examples include the #WeAreStillIn and America’s Pledge initiatives. In both cases, the idea was to reaffirm the fact that despite Trump’s decision, the USA, through the combined efforts of business leaders, university chancellors, mayors and state governors, would fulfil—and even surpass—its Paris commitments. In addition to mobilising non-state and sub-state actors, the priority was also to find a new ‘climate champion’ and saviour of multilateralism to fill in the gap created by the US exit. Despite his status as relative newcomer to the climate cause, French president Emmanuel Macron was rapidly elevated to the rank of ‘champion of the earth’. The organisation of a press conference at the Elysée Palace the day after Trump’s announcement in June 2017 and the hosting of the One Planet Summit in December 2017 were coordinated efforts to retain control of the overall climate narrative and through this, keep the ‘Paris prophecy’ alive. In our view, these and other concerted efforts to ‘save’ the Paris agreement and ‘ramp up ambition’, by being almost exclusively centred on the production of narratives and signals, pose the risk of further ‘virtualising’ global climate governance (Carrier and West 2009). Moreover, the Paris approach’s ‘performative’ dimension complicates the task of publicly recognising that targets—such as the 1.5 °C target—are out of reach (Geden 2015a). Faced with the need to uphold a positive storyline, stakeholders of global climate governance are incentivised to ‘move the goal posts’ through ‘creative accounting’ or unproven techno-fixes, as exemplified by the massive amounts of ‘negative emission technologies’ included in global decarbonisation scenarios (Anderson 2015; Geden 2015b). By doing so, they risk delaying the necessary acknowledgement that current modes of development are inherently unsustainable.

### Uneven political geographies of global regulation

In other words, there is a real danger of deepening the rift between an ‘international community’ seemingly committed to ambitious climate action and the reality of ‘business as usual’ in a rapidly warming world. This discrepancy is not unique to the current period. The last decades saw a growing disconnect, or ‘schism’ (Aykut and Dahan 2015; Aykut 2016), between, on the one hand, a slow and procedural UN arena focused on negotiating carbon emission reductions, and on the other hand, a staggering acceleration of a series of phenomena that are at the heart of the climate crisis but outside of climate governance’s remit. Chief among these are the dynamics of economic and financial globalisation, the expansion of extractivist development models and the global spread of Western consumerist lifestyles. Indeed, ‘climate policy’ is an inherently crosscutting policy domain. It touches on a range of very different issues, from development and energy policy, to trade and financial regulation, as well as agriculture and urban planning. Yet, the governance of these issues follows very different logics.

The voluntary and soft-law approach to climate governance contrasts with the situation in other issue areas. Some of these are regulated through ‘hard law’, enforced by international organisations, while others are exempt from global regulation, and governed instead through global market dynamics and power relations (Kingsbury 2011). Each governance arrangement draws on specific tools and mechanisms to exert influence on relevant actors and practices. The shift in global climate governance brought about through the Paris agreement has exacerbated these differences. Indeed, while it is very ambitious in terms of its global temperature targets, the Paris agreement is evasive when it comes to spelling out the changes that will be required to attain them. There is no mention, for instance, of phasing out fossil fuels or ‘decarbonising’ the global economy, nor, for that matter, of encouraging renewables or energy efficiency. Another important issue that is completely absent from the text is international trade regulation (Brandi et al. 2015). This links back to the ‘fragmentation’ of global governance, whereby the management of a problem falls upon diverse international organisations with potentially contradictory objectives (Biermann et al. 2009). These fragmentations owe nothing to chance but are rather the product of structural ‘selectivities’ that are rooted in the global order and protected by powerful interests (Brunnengräber 2013). Saudi Arabia and other fossil fuel interests, for instance, actively worked to prevent any discussion on energy questions within the Climate Convention, so as to thwart any international regulation in that domain (Aykut and Castro 2017; Depledge 2008). The same applies to trade, whose absence from the climate negotiations is due to the active efforts of a coalition of industrialised and emerging economies (Luterbacher and Norrlöf 2001). It is worth noting, however, that the similarities between the two cases stop there. Unlike energy, international trade is regulated through a fairly robust international organisation, the World Trade Organisation (WTO) and a number of legally binding bilateral treaties (Mattli and Woods 2009). From ‘non-governed’ issues where the strongest get their way (such as energy), to issues that are regulated through legally binding treaties (such as trade), to those managed through *soft law* (such as human rights and most environmental issues), we are in the midst of an increasingly complex global governance landscape. This landscape is not set in stone but is the product of political strategies and historical struggles that continue to act as barriers to an effective management of the climate crisis. The multi-dimensional nature of the problem calls for an in-depth rethinking of the established global order, beginning with the existing division of labour and hierarchy between international organisations, and the regulatory void when it comes to strategic domains such as fossil fuel production and trade.

## Concluding remarks

Scholars of international relations generally agree that a central feature of international regimes is that actors’ expectations converge in a given area of international relations (Krasner 1983). While it has generally been thought that such convergence is best reached through binding regulations and the building of strong international organisations, this no longer holds for Paris-type governance arrangements. A growing body of scholarship therefore examines the Paris shift and considers its consequences. In this article, we argued for the need to broaden the perspective adopted in this literature along two broad lines. We first suggested to re-embed the voluntary turn in climate governance within broader transformations in the ways that global problems are governed. In the course of these transformations, neo-managerial tools and techniques are increasingly adopted by international organisations. Second, we argued that the new climate governance is not only characterised by institutional innovations. It also builds on narratives and signals as central means of implementation, by aligning actors’ expectations and coordinating their behaviour towards a low-carbon future. Based on these observations, we suggest the term *incantatory governance* to characterise the Paris framework. The term highlights the iterative nature of the new ‘bottom-up’ and voluntary governance process. It also points to the increasing prominence of communicative devices and marketing techniques in global climate governance. Our analysis suggests that both of these evolutions—the rise of ‘soft law’ approaches and the widespread deployment of communicative techniques—reflect a much broader process: the entry of management culture, techniques and actors into global environmental governance.

Having said this, we consider the analyses laid out in this article as no more than a starting point. We hope that they will inspire further research on the discursive and performative dimensions of the new climate governance, but also beyond. Indeed, given the climate arena’s central position in global politics, one can expect other governance spaces to draw inspiration from it. This makes it all the more important to scrutinise the mechanisms of post-Paris climate governance, evaluate their effectiveness, signal potential drawbacks and understand the governance shift’s wider implications. One final observation should be made relating to the challenges facing those who express more fundamental reservations about the brave new world of ‘performative’ or, as we have termed it, ‘incantatory’ governance. Critical perspectives are important in order to both problematise the selective and fractioned geographies of global regulation and highlight the shortcomings of a climate governance architecture that brushes aside issues that are key to solving the problem. And yet, there is little room for radical or fundamental critique under the current climate governance since such critique risks undermining the ‘Paris prophecy’ by sending negative ‘signals’. How then can we avoid both complacent self-censorship and a sterile, and potentially destructive, critical stance? We can perhaps begin by recognising that in a fractured and divided world, and in the face of multiple and interrelated crises, the Paris agreement provides a snapshot of what can presently be expected from the UN system and the UNFCCC process. It therefore goes without saying that the climate problem cannot be solved within the UN system alone and that the Paris agreement only forms one piece of a much larger puzzle. Solving this puzzle will require actions at multiple levels and in a wide range of arenas. While this includes businesses, it also encompasses states and regulations, other international organisations, as well as collective mobilisations and social movements, which appear as key to shifting current power relations in favour of transformative change. It will also involve long and arduous efforts to re-politicise the climate debate and show the connections between climate change and other important issues that have traditionally been ignored in, or excluded from global climate governance. With their own rituals, heroes and discourses, recent and innovative climate protests, from ‘Fridays for future’ to Extinction Rebellion, can be interpreted as attempts to do just that.

Our concluding remark is inspired by the current situation of global confinement and lockdown in the struggle against the CoVid-19 virus. In many countries, and especially of the Global North, the measures imposed by governments to address this global health crisis are unprecedented since World War II. The command-and-control approach combining quarantines, curfews and emergency laws stands at the antipodes of the managerial and ‘incantatory’ governance approach that we just analysed. It is too soon to say how this crisis and its political and economic consequences will affect the prospects of global decarbonisation. However, the contrast between these two governance models—one centred on transnational coordination through signals and narratives, the other on command-and-control and the sovereign power of nation-states—is striking. It could well be, therefore, that the CoVid-19 experience deeply affects and transforms, yet again, the discursive context of climate governance.

## Footnotes

1 See, for instance, the special forum section ‘Reflections on the Paris Agreement on Climate Change’ in *Global environmental politics* (2017, Vol. 15, No. 3).

2 E.g. the special issues in *Climate Law* (2016, Vol. 6, No. 1–2) and *Climate Policy* (2017, Vol. 17, No. 1).

3 The transparency framework is further spelled out in the ‘Katowice Rulebook’ adopted at COP24 in 2018.

4 For an overview, see Aykut (2016).

5 https://climateaction.unfccc.int/views/about.html (accessed 9 September 2019).

6 Cited in Losson, Christian, ‘COP21: “L’accord doit être une prophétie autoréalisatrice”’, *Libération*, 17.12.2015.

7 As the Copenhagen Accord faced fierce resistance from a number of developing countries and failed to be formally adopted by the COP, it was only at the 2013 Warsaw summit that INDCs were officially adopted (https://unfccc.int/resource/docs/2013/cop19/eng/10a01.pdf#page=3, accessed 9 September 2019).

8 An informal GoogleGroup set up in the lead-up to Paris and that brings together key non-state actors involved in or around the UNFCCC process.

9 https://www.climatechangenews.com/2019/09/16/net-zero-story-target-will-shape-future/, accessed 12 March 2020.

10 https://ciff.org/grant-portfolio/climate-briefing-service/ (accessed 9 September 2019).

11 We Mean Business unites numerous partners, including the World Business Council for Sustainable Development, Business for Social Responsibility, CDP Worldwide, The B-Team, The Climate Group, The Prince of Wales’ Corporate Leaders Group and the Coalition for Environmentally Responsible Economies.

12 Cited in Goodell, Jeff, ‘Will the Paris Climate Deal Save the World?’ *Rolling Stone*, no. 1253, 28.1.2016.

13 https://www.bloomberg.com/news/articles/2020-02-26/behind-europe-s-green-deal-a-quiet-campaign-by-hidden-powerbrokers (accessed 7 March 2020).
